# The Parotid Puzzle: A Case of Juvenile Recurrent Parotitis

**DOI:** 10.7759/cureus.84786

**Published:** 2025-05-25

**Authors:** Nusrath M P, Mitra A Ghafoor Zeyaei, Abdul Razaq Yousefi, Lina Anwar

**Affiliations:** 1 Pediatric Emergency Medicine, Al Jalila Children's Speciality Hospital, Dubai, ARE; 2 Medical Education, Brunel Medical School, London, GBR

**Keywords:** children, juvenile recurrent parotitis, parotid gland, swelling, ultrasonography

## Abstract

Juvenile recurrent parotitis is characterized by recurring, non-obstructive, non-suppurative parotid inflammation in children between the ages of one and 16 years. Usually occurring between the ages of three and five, this condition typically goes away around adolescence. It is an uncommon condition, and the cause of this rare illness is unknown, however it is most likely multifactorial. Although the swelling of the parotid tends to be unilateral, it can occur bilaterally, with one side being more affected than the other.

We report a case of a five-year-old girl who had five episodes of recurrent parotid swelling. We present this case with the objectives of highlighting the symptoms and signs of this uncommon illness and emphasizing the importance of ultrasonography as a diagnostic tool.

## Introduction

Juvenile recurrent parotitis (JRP) is the term used to describe recurring inflammatory parotitis in children that has no clear cause. It is an uncommon disorder and characterized by multiple episodes of parotid swelling. It commonly presents with pain, swelling and fever of less than one week duration and there will be asymptomatic periods between episodes [1]. Most of the time, symptoms resolve spontaneously after puberty. It indicates that salivary duct constriction is a contributing factor; as the child grows the duct expands and the flow of saliva returns to normal. In children, up to 10% of all salivary gland disorders are caused by sialadenitis. After the mumps, JRP is the second most frequent cause of childhood parotitis.

Although it can happen on both sides, this pathology is typically unilateral, with symptoms typically being more noticeable on one side. The current consensus supports a multifactorial origin for JRP, although the pathogenesis is still unknown. Congenital ductal abnormalities, hereditary genetic variables, bacterial or viral infections, allergies, and localized manifestations of autoimmune diseases are some of the causes that have been proposed as contributing to the development of JRP. However, the primary explanation for the pathophysiology of JRP is thought to be a decrease in salivary production and a lack of salivary outflow through the ductal system, which promotes infections of the ascending salivary glands through the oral cavity. Retention-induced partial obstruction is gradually followed by duct dilatation, which further facilitates infection. Our goals in presenting this case are to draw attention to the symptoms and indicators of this rare disease and to underscore the value of ultrasonography as a diagnostic method.

Bacterial or viral infections, autoimmune diseases such as Sjögren's syndrome, and lupus are other possible causes of parotitis [2,3]. Other viruses have been linked to the causes of parotitis in addition to mumps, including Epstein-Barr virus (EBV), parainfluenza, adenovirus, human herpes virus type 6 (HHV-6), hepatitis C virus, and HIV.

The incidence has a bimodal onset age, peaking between the ages of nine to 11 and three to six years [4]. Obstructive lesions, dental malocclusion, Sjögren syndrome, and IgA deficiency were proposed as exclusion criteria by Garavello et al. [5]. Age <16 years, recurrent unilateral or bilateral painful parotid swelling, and at least two episodes during the previous six months were proposed as inclusion criteria. The clinical picture serves as the basis for the diagnosis of JRP, which ultrasonography can confirm. Multiple hypoechoic zones with vacuolization inside the parenchyma are visible on ultrasound [6]. Although there is currently no standard treatment, the goal of using analgesics and antibiotics during acute episodes is to reduce symptoms and avoid harming the gland parenchyma.

## Case presentation

A five-year-old previously healthy girl had multiple episodes of painful swelling of the left parotid gland, five episodes in 1.5 years. Each episode, the swelling lasted for five to seven days. She had received vaccinations up to her age, including measles, mumps, rubella (MMR). There was no history of mouth and eye dryness, joint pains or swelling, skin rashes, or weight loss. During the first episode she was diagnosed with mumps. In the family, there is no history of recurrent parotid swelling or autoimmune disorders. On examination the child was afebrile, and vitals were within normal limits. Further, the left parotid gland was enlarged, firm, tender, and mildly erythematous. Neither xerostomia nor xerophthalmia was evident. The intraoral exam showed erythema around the Stensen's duct (parotid duct) opening; pressure over the gland did not cause any purulent discharge from the duct. Other systemic examinations were unremarkable. 

Blood count, inflammatory markers, renal function tests, and serum electrolytes were within normal limits except for mildly elevated CRP, which suggests inflammation. Mumps antibodies done during the first episode showed immunity, and IgM was negative, suggesting serological test for mumps was negative (Table 1).

**Table 1:** Full blood count MCV: mean corpuscular volume; MCH: mean corpuscular hemoglobin; MCHC: mean corpuscular hemoglobin concentration; RDW: red cell distribution width; MPV: mean platelet volume; ESR: Erythrocyte sedimentation rate

COMPONENT	REFERENCE RANGE	RESULT
WBC count	5.0 - 15.0 10^3/uL	12.8
RBC count	4.00 - 5.20 10^6/uL	5.28
Hemoglobin	11.1 - 14.1 g/dL	10.7
Hematocrit	30.0 - 38.0 %	34.6
MCV	72.0 - 84.0 fL	65.5
MCH	25.0 - 29.0 pg	20.3
MCHC	31.5 - 34.5 g/dL	30.9
RDW	11.5 - 14.0 %	15.2
Platelet count	200 - 490 10^3/uL	375
MPV	7.4 - 10.4 fL	10.3
Neutrophil absolute	1.5 - 8.0 10^3/uL	6.4
Lymphocytes absolute	6.0 - 9.0 10^3/uL	4.8
Monocyte absolute	0.20 - 1.00 10^3/uL	1.5
Eosinophil absolute	0.10 - 1.00 10^3/uL	0.1
Basophil absolute	0.00 - 0.10 10^3/uL	0.1
Neutrophil %		49.9
Lymphocyte %		37.6
Monocyte %		11.3
Eosinophil %		0.7
Basophil %		0.5
C-Reactive Protein	0 - 5 mg/L	34.2
ESR	0 - 20 mm/1hr	10

**Figure 1:**
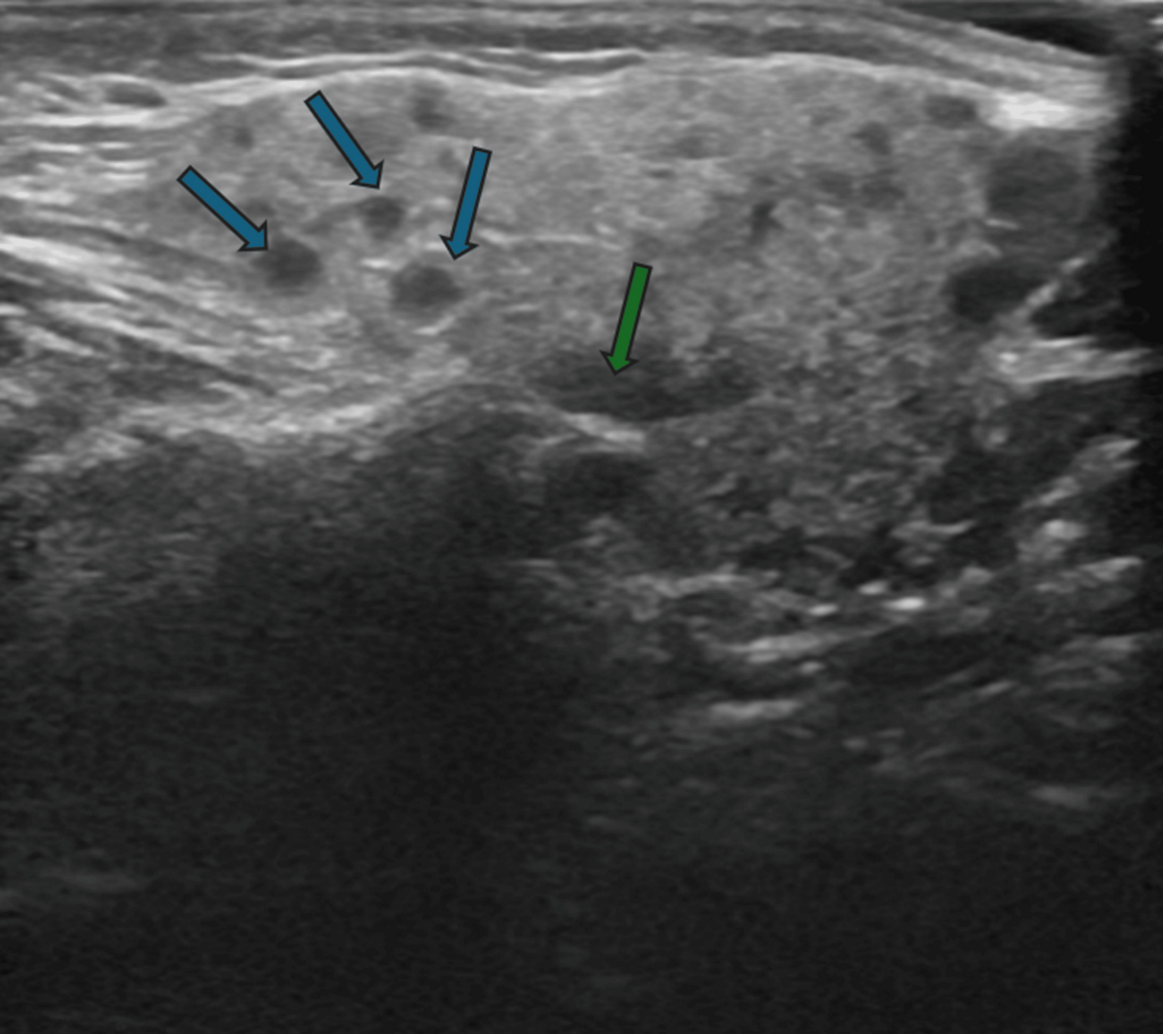
Left parotid gland is mildly bulky and hypoechoic. Multiple small intraparotid lymphadenitis noted. Blue arrow- Hypoechoic areas; Green arrow- Intraparotid lymph nodes

Ultrasound done in the last episode showed the left parotid gland is bulky (2.5 cm depth) and mildly hypoechoic. Evidence of multiple intraparotid lymphadenitis was noted and measured at 1.2 cm. There was no evidence of necrosis/abscess formation. There was no evidence of parotid duct dilatation/calcification. The left submandibular gland was normal in size and echotexture. Evidence of multiple small left upper cervical lymphadenitis was noted, the largest one noted at the left submandibular region measuring 1.5 cm. There was no evidence of necrosis/abscess formation. The right side parotid gland was normal size and normal echotexture. No focal lesions were seen and no duct dilatation (Figure 1).

**Figure 2:**
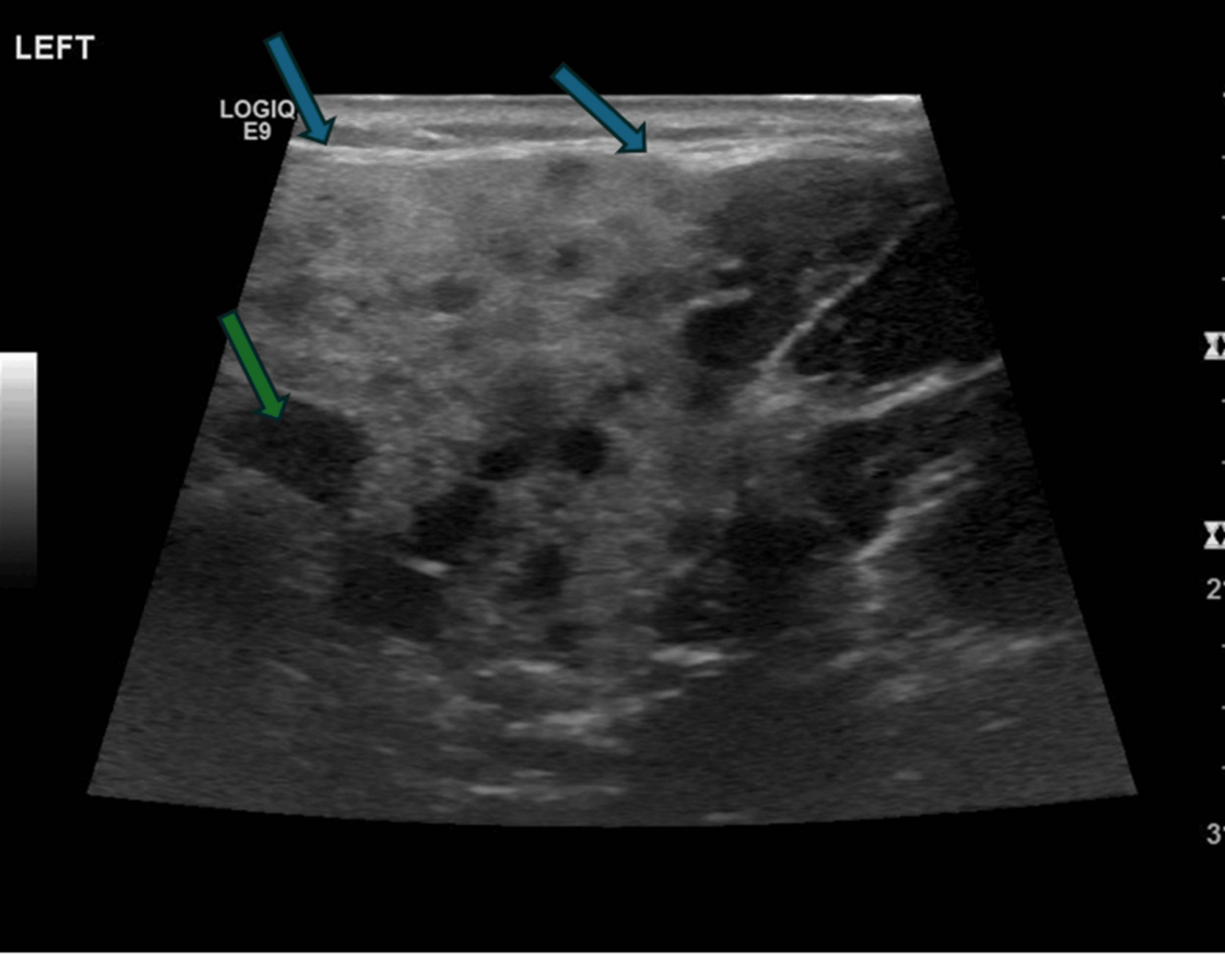
Diffuse swelling of the left parotid gland parenchyma with increased echogenicity. Glandular parenchyma shows multiple different sizes intra parotid lymph nodes. No features of abscess formation. Blue arrow- Increased echogenicity of left parotid gland parenchyma; Green arrow- Intraparotid lymph node

Ultrasound during the first episode showed diffuse swelling of the parotid gland. The glandular parenchyma was heterogeneous and mixed with hyperechoic and hypoechoic areas. There were no features of abscess formation. There were no features of ductal dilatation or features of calcification or calculus formation. There were mildly enlarged intraparotid lymph nodes. The right parotid gland appeared normal. Usual lymph nodes were identified in the neck. Sonographic features were consistent with left parotitis (Figure 2).

Analgesics and oral antibiotics were used to treat each episode, which resolved in five to seven days. Juvenile recurrent parotitis was diagnosed in view of the recurrent episodes of parotitis. The child was seen in the ENT clinic, and it was explained to the parents that it is a self-limiting condition, and the plan was to consider sialendoscopy if there is a recurrence of severe episodes. She remained asymptomatic for the last one year.

## Discussion

A rare recurrent inflammation of the parotid glands that affects youngsters is called juvenile recurrent parotitis. It presents as bilateral or unilateral parotid swelling recurring at least twice before puberty with eventual termination in the second decade of life [7,8]. Episodes often appear between the ages of three and six years, with a male majority, last one day to two weeks, and happen every three to four months. Most patients experience a spontaneous resolution during puberty [7,8]. Our case child was five years old, and she had episodes every two to three months; each episode resolved in five to seven days. In our case, the parotid gland enlargement is unilateral; as per Papadopoulou-Alataki et al. [9] and Leerdam et al. [10], this occurs in 66% and in 74% of cases, respectively. In rare cases, the gland enlargement may be bilateral, with one side being more affected than the other [9]. Clinically, the swelling is unpleasant and is followed by either a localized or systemic rise in body temperature, as well as localized erythema. Each patient experiences attacks at different frequencies and intervals [9]. Although the exact cause of JRP is unknown, congenital parotid gland malformations that result in retrograde infections, allergies, immunodeficiencies, and autoimmune diseases, primarily Sjögren's syndrome (SS) or sarcoidosis, have been proposed as causes [11]. The pathophysiology of JRP is thought to be complex, even though it is not entirely understood. One explanation could be decreased salivary flow or altered salivary content, which causes stasis, repeated retrograde inflammation, and ductule destruction, ultimately leading to the development of sialectasis. According to a different theory, recurring inflammation is mostly caused by baseline anatomical abnormalities of the gland ductules [10,12]. Histologically, there is sialectasis, or intraductal cystic dilatations of peripheral ducts, and periductal lymphocytic infiltrate. Ectatic ducts are usually 1-2 mm in diameter and resemble a white ductal layer without the healthy blood vessel covering it, in contrast to a normal gland. This feature is thought to be typical of JRP [7]. 

In terms of differential diagnosis, the following conditions involving a parotid swelling should be ruled out: congenital cystic lesion, sialolithiasis, mumps, mandibular osteomyelitis, lymphoepithelial cyst, primary SS, benign tumors and malignancies (leukemia, lymphoma) [13]. In our case, the initial swelling event that was identified as mumps could have been an early manifestation of JRP; nevertheless, the investigation did not support mumps. It is difficult to distinguish between the first episode of JRP and the mumps. One significant distinction between JRP and mumps is that a kid with mumps experiences fever, discomfort, headaches, and chills; JRP symptoms are typically more localized in the parotid gland and include occasional fever episodes [7]. Initially diagnosed clinically as mumps; however, laboratory confirmation was not conclusive, and later episodes confirmed a pattern consistent with JRP. The most common signs of SS are parotid enlargement and malfunction of the salivary and lacrimal glands. Recurrent parotitis, especially when bilaterally involved, seems to be the most prevalent symptom in children with SS, albeit these symptoms are not always present simultaneously [9,13]. 

The recurrence of the parotid swelling triggers the JRP diagnosis. With at least two episodes of acute parotitis, the diagnosis of JRP is mostly made based on the patient's medical history and physical examination [14]. Ultrasonography is commonly used to confirm the diagnoses after ruling out other potential causes, including acute infectious parotitis, sialoliths, ductal malformation, malignancies and systemic allergy or autoimmune illnesses [9,15]. Previously, sialography was used to diagnose sialectasis; however, ultrasound has now replaced this method [10]. An enlarged and heterogeneous parotid gland, involving either one or both sides, as well as hypoechogenic regions 2-4 mm and/or hyperemia on Doppler ultrasound, which are suggestive of sialectasia or lymphocytic infiltration, are common ultrasonography findings [15]. For children, ultrasonography is the best imaging modality because it is noninvasive. In JRP, dispersed hypoechoic foci (known as "Swiss cheese" or "moth-eaten") are expected ultrasonography findings [16]. Compared to ultrasonography, Quenin and associates discovered that sialendoscopy was more sensitive [17]. A more recent technique for diagnosing disorders of the salivary glands is sialendoscopy. Sialendoscopy helps in the diagnosis of JRP by visualizing strictures, hypovascularization, and whitish intraductal debris and it has a concurrent therapeutic effect. The following table describes a comparison between sialography, ultrasonography and sialendoscopy (Table 3).

**Table 2:** A comparison of ultrasonography, sialography and sialendoscopy JRP- Juvenile Recurrent Parotitis

Imaging	Ultrasonography	Sialography	Sialendoscopy
Procedure	Non-invasive imaging modalities use high frequency sound waves to create images.	Injecting a contrast medium into the salivary ducts and subsequent X- ray imaging. Visualization of the anatomy of the ductal system	Minimally invasive technique, inserting a small flexible endoscope into salivary duct through oral cavity. This gives direct visualization of inner ductal system.
Findings	Multiple hypoechoic spots measuring (2-4 mm) within the gland, which are dilated ducts (sialectasis). Intraglandular enlarged lymph nodes.	Establish the diagnosis by visualization of punctate sialectasis and peripheral duct dilatation, producing a sausage-like appearance.	Direct visualization of salivary duct helps identify various abnormalities such as mucus plugs, fibrinous debris, ductal stenosis , and abnormal appearance of the ductal wall. These are characteristic of JRP.
Advantages	1. Preferred initial modality 2. Non-invasive 3. Lack of radiation 4. Wide availability 5. Help monitoring the changes in the gland overtime	1. Therapeutic- Due to the flushing effect of the contrast medium, studies have proven a significant reduction in the incidence post sialography. 2. Simplicity of the procedure 3. Performed in an outpatient setting 4. May be repeated as necessary	1. Therapeutic- Saline irrigation of the duct may be employed to remove mucus plugs and inflammatory debris 2. Balloon dilation can be performed to treat ductal strictures. 3. Medications like corticosteroids and antibiotics can be instilled directly into the duct. 4. Reducing the frequency and severity
Limitations	1. The diagnostic accuracy is operator dependent. 2. Might not always visualize the deeper structures of the gland as clearly as other modalities	1. Invasive procedure 2. Exposure to ionizing radiation 3. Risk of allergic reactions to the contrast medium	1. Requires anesthesia 2. Limited availability

Both conservative and invasive surgical methods are available as treatment options. Acute symptom alleviation is typically the goal of JRP treatment. Using painkillers, maintaining proper dental hygiene, drinking enough water, and massaging the parotid gland warmly are the initial steps [10]. Use of chewing gum and sialagogic substances are beneficial. The administration of antibiotics during attacks is controversial as this condition is rarely purulent [11]. Antibiotics and analgesics are really used to treat acute episodes in order to reduce symptoms and stop further harm to the glandular parenchyma [18]. Nevertheless, there is no proof that antibiotics have an impact on how long episodes last [8]. In our case, analgesics and oral antibiotics were used to treat each episode, which resolved in five to seven days. More active therapy may be taken into consideration when the frequency and severity of the swelling episodes are extreme, interfering with a child's social and academic life and increasing the likelihood of serious health issues. Furthermore, the few patients who experience aftereffects such as persistent swelling, recurrent pain, and decreased gland function are candidates for further invasive treatment.

Sialendoscopy is a surgical procedure in which the saliva duct is examined with a tiny fiberoptic camera. Examining the interior of the ducts helps confirm the diagnosis of juvenile recurrent parotitis. For the treatment of such severe cases of JRP we use a small inflatable balloon which enlarges the duct. Other methods include cleaning or rinsing the ducts with saline solutions using sialendoscopy.

These treatments aid in restoring normal salivary flow and averting recurring parotitis outbreaks. The endoscope can occasionally be used to deliver steroids as well. Nahlieli et al. used sialendoscopy to perform duct probing with lavage, dilation, and hydrocortisone injection; the symptoms resolved, and the follow-up showed a very low recurrence rate [7]. Sialendoscopy and lavage with saline solutions or corticosteroids were very successful and had a very low recurrence rate, according to a meta-analysis by Canzi et al. [19]. Only patients with ongoing problems should receive more aggressive treatment, which involves parotid duct ligation, parotidectomy, or tympanic neurectomy, all of which have disappointing outcomes [20].

Ninety percent of patients experience a natural resolution of their symptoms by puberty and don't require invasive treatment. In a small number of severe cases, the glandular parenchyma is destroyed, and its functionality is reduced by 50% to 80% [10]. Long-term results are usually favorable, even in more severe cases that require invasive treatment. Pediatricians should be knowledgeable about unilateral or bilateral parotid edema and its treatment choices. Children with this illness need to be monitored frequently and managed early.

## Conclusions

When a child experiences multiple episodes of parotitis, JRP should be taken into consideration. The primary diagnosis is clinical, and ultrasonography is used to confirm it. Ultrasonography is non-invasive and readily available imaging modality for the initial evaluation of suspected JRP. This is usually a self-limiting condition, and episodes resolve by puberty. With prompt diagnosis, JRP can be appropriately managed conservatively. When treating a pediatric patient who exhibits recurrent parotid edema, the doctor must always take JRP into account as a differential diagnosis. Evaluation for underlying rheumatologic diseases or immune defects should be considered.

Effective management necessitates collaboration among healthcare professionals, including pediatricians for initial assessment and ongoing care, radiologists for accurate image interpretation, and potentially otolaryngologists for advanced interventions like sialendoscopy in refractory cases.
